# Dietary Patterns and Sustainable Lifestyles: A Multicenter Study from Latin America and Spain †

**DOI:** 10.3390/foods14122065

**Published:** 2025-06-11

**Authors:** Solange Parra-Soto, Tannia Valeria Carpio-Arias, Israel Rios-Castillo, Patricio Pérez-Armijo, Leslie Landaeta-Díaz, Ana Gabriela Murillo, Jacqueline Araneda-Flores, Brian M. Cavagnari, Georgina Gómez, Gladys Morales, Karla Cordón-Arrivillaga, Melissa Miranda-Durán, Ana María Aguilar, Alfonsina Ortiz, Eliana Romina Meza-Miranda, Edna J. Nava-González, Jhon Jairo Bejarano-Roncancio, Beatriz Núñez-Martínez, João P. M. Lima, Jorge de Assis Costa, Jairo Torres, Saby Mauricio, Saby Camacho, Gloria Maricela Morales, Macarena Jara, Samuel Durán-Agüero

**Affiliations:** 1 Departamento de Nutrición y Salud Pública, Facultad Ciencias de la Salud y de los Alimentos, Universidad del Bío-Bío, Chillán 3780000, Chile; sparra@ubiobio.cl (S.P.-S.); jaraneda@ubiobio.cl (J.A.-F.); 2 Grupo de Investigación en Alimentación y Nutrición Humana (GIANH), Facultad de Salud Pública, Escuela Superior Politécnica de Chimborazo, Riobamba 060104, Ecuador; tannia.carpio@espoch.edu.ec; 3 Organización de las Naciones Unidas para la Alimentación y la Agricultura (FAO), Oficina Subregional de la FAO en Mesoamérica, Panamá 0843-00006, Panama; israel.rios@fao.org; 4 Escuela de Nutrición y Dietética, Facultad de Medicina, Universidad de Panamá, Panamá 0843-00006, Panama; 5 Facultad de Ciencias de la Salud, Universidad Isabel I, 09003 Burgos, Spain; patricioesteban.perez@ui1.es; 6 Escuela de Nutrición y Dietética, Facultad de Salud y Ciencias Sociales, Universidad de Las Américas, Santiago 8330015, Chile; llandaeta@udla.cl; 7 Departamento de Bioquímica, Escuela de Medicina, Universidad de Costa Rica, Montes de Oca, San José 2060, Costa Rica; gmurillo.nutricion@gmail.com (A.G.M.); georginagomez.ucr@gmail.com (G.G.); 8 Facultad de Ciencias Médicas, Pontificia Universidad Católica Argentina, Av. Alicia Moreau de Justo 1300, Buenos Aires C1107, Argentina; bcavagna@gmail.com; 9 Departamento Salud Pública, Facultad de Medicina, Universidad de La Frontera, Temuco 4780000, Chile; gladys.morales@ufrontera.cl; 10 Unidad de Investigación en Seguridad Alimentaria y Nutricional, Escuela de Nutrición, Facultad de Ciencias Químicas y Farmacia, Universidad de San Carlos de Guatemala, Guatemala 01012, Guatemala; krcordon@gmail.com; 11 Instituto de Investigación en Salud y Desarrollo, Universidad Mayor de San Andrés, La Paz, Bolivia; melm2m16@gmail.com (M.M.-D.); ana.aguilar@umsalud.edu.bo (A.M.A.); 12 Licenciatura en Nutrición, Facultad de Ciencias de la Salud, Universidad Católica del Uruguay, Montevideo 11600, Uruguay; alortiz@ucu.edu.uy; 13 Centro Multidisciplinario de Investigaciones Tecnológicas, Universidad Nacional de Asunción, Campus San Lorenzo, San Lorenzo 2160, Paraguay; mezamirandaelianaromina@gmail.com; 14 Facultad de Salud Pública y Nutrición, Universidad Autónoma de Nuevo León, Monterrey 64460, Nuevo León, Mexico; edna.navag@uanl.mx; 15 Departamento de Nutrición Humana, Facultad de Medicina, Universidad Nacional de Colombia, Bogotá 111321, Colombia; jjbejaranor@unal.edu.co; 16 Coordinación General de Investigación, Universidad del Norte, Asunción 001218, Paraguay; benunez@uninorte.edu.py; 17 H&TRC—Health & Technology Research Center, Coimbra Health School, Polytechnic University of Coimbra, 3045-043 Coimbra, Portugal; joao.lima@estesc.ipc.pt; 18 GreenUPorto—Sustainable Agrifood Production Research Centre, 4485-646 Vairão, Portugal; 19 SUScita—Research Group on Sustainability, Cities and Urban Intelligence, 3045-093 Coimbra, Portugal; 20 Departamento de Ciências Humanas e Linguagens (DCHL), Universidade do Estado de Minas Gerais (UEMG), Núcleo de Estudo e Pesquisa em Educação e Saúde (NEPES), Ubá 36500-000, Brazil; nyron32@gmail.com; 21 FoodChemPack Research Group, Department of Analytical Chemistry, Nutrition and Food Science, Faculty of Pharmacy, University of Santiago de Compostela, Campus Vida, 15782 Santiago de Compostela, Spain; jairoalonso.torres@rai.usc.es; 22 Programa Académico Nutrición y Dietética, Facultad de Ciencias de la Salud, Universidad Privada Norbert Wiener, Lima 15000, Peru; saby.mauricio@uwiener.edu.pe; 23 Academia AMIR México, Ciudad de Mexico 03100, Mexico; sabycamacho@gmail.com; 24 Escuela de Medicina y Ciencias de la Salud, Tecnológico de Monterrey, Ciudad de Mexico 14380, Mexico; 25 Instituto Salvadoreño de Bienestar Magisterial (ISBM), Policlínico de San Salvador y Centro de Hemodiálisis de El Salvador, 503 San Salvador, El Salvador; maricela4264@hotmail.com; 26 Escuela de Nutrición y Dietética, Facultad de Ciencias de la Rehabilitación y Calidad de Vida, Universidad San Sebastian, Providencia 7500000, Chile; mjaran@docente.uss.cl

**Keywords:** dietary pattern, vegan diet, vegetarian diet, Western diet, Mediterranean diet, sustainable lifestyles

## Abstract

Food systems interact through multiple dimensions including food security, nutrition, and planetary health. This study aims to associate different dietary patterns with sustainable lifestyles in Latin America and Spain. This was an observational, analytical, multicenter, cross-sectional survey study, with a total of 6412 participants. A self-administered questionnaire was developed in an online format in the Google Docs interface. The questionnaire was divided into sections: (1) sociodemographic background: country of residence, age, sex, educational level, socioeconomic aspects, and place of residence; (2) body mass index classification; (3) dietary patterns (Western, vegetarian, vegan, ketogenic, Mediterranean, prudent, or paleolithic diets); and (4) the Sustainable Lifestyles Survey. Multivariate models were applied to adjust for potential confounding factors. The mean age of the participants was 35.2 years (SD 12.7). The majority of participants identified their dietary pattern as omnivorous (41.5%), followed by the Western diet (21.7%) and the Mediterranean diet (12.7%). Plant-based, vegan (β: 14.90; 95% CI: 9.75–20.05), and lacto egg (β: 12.08; 95% CI: 8.57–15.58) diets are significantly associated with a higher sustainability score compared to an omnivorous diet. In contrast, a Western diet is inversely associated (β: −5.63; 95% CI: −7.20 to −4.06). Finally, a vegan (Sub-score 1: β: 6.19; 95% CI: 4.43–7.96) diet is consistently associated with higher levels of sustainability in all areas assessed. In contrast, the Western diet shows a significant negative association with sustainability in all subcomponents assessed. Conclusions: Plant-based dietary patterns were shown to be associated with sustainable lifestyles, with the vegan diet having the greatest association, while the Western dietary pattern was inversely associated.

## 1. Introduction

Food systems coexist at the intersection of several factors such as food security, nutrition, individual and population health, planetary health, and social justice, among others [1,2,3]. Climate change causes adverse effects on human health [4], as the increased frequency and intensity of climatic events affect food availability and livelihoods, as well as the nutritional content of food, putting populations at risk of nutritional deficiencies [5]. In addition, the food system contributes to soil erosion, air and water pollution, and is responsible for one-third of all greenhouse gas emissions, contributing to global warming [6,7,8,9].

The Food and Agriculture Organization of the United Nations (FAO) defines healthy and sustainable diets as “those with a low environmental impact, which contribute to food and nutritional security and a healthy life for present and future generations” [10], which is in line with previous definitions of sustainable consumption [11]. More recently, a report by the FAO and WHO states that healthy diets should be adequate, balanced, include a wide dietary diversity, and moderate the consumption of products with a high content of nutrients that are critical for human health [12]. Moreover, the EAT-Lancet Commission proposes a healthy diet based on sustainable food systems [10], identifying three main areas for its transformation: improvements in production, widespread change in dietary patterns, and reduction of waste. However, to date, there is no widely accepted, operational definition of sustainable food consumption behaviors, and the associated factors remain unclear [13].

In 2021, the United Nations made a global call to think about the role of food systems in the sustainable development agenda [14,15]. This is the World Summit on Food Systems [15], which proposes several actions, especially the development of national roadmaps for the transformation of food systems towards more sustainable models that will make it possible to achieve the Sustainable Development Goals (SDGs) [16].

In Ibero-America, more than 10 countries have roadmaps for this purpose. However, the lack of evidence on this matter could be a limitation that hinders the implementation of these roadmaps.

The current food system is characterized mainly by being industrial and globalized: although it has managed to increase food production in the world, it is also associated with the generation of negative impacts that are also notable, with important environmental, social, and health consequences for the population [17].

The negative effects of the current food system include the devaluation of agriculture, land concentration, loss of biodiversity, contamination of groundwater, and the impoverishment of rural and peasant communities due to the loss of their livelihoods, among others. International food trade has aggravated environmental contamination resulting from packaging and transportation, affecting the environment and harming small- and medium-sized producers [18].

What we eat has a major impact on our health and the health of the planet. Western-style omnivorous diets in countries with high and upper-middle income populations generally include large amounts of animal foods, a high energy intake, and a high intake of saturated fats, salt, sugar, refined grains, and oils, usually higher than recommended, with a predominance of highly industrialized and processed products. In addition, the increase of production, availability, promotion, and consumption of ultra-processed, low-cost, highly palatable products, with a high caloric density and high content of critical nutrients, favor the development of obesity and related co-morbidities [4,19,20].

The 2015 Paris Agreement on climate change provided a response to the multiple challenges linking nutrition and climate change, highlighting the need for healthy and sustainable diets that help ensure the health of the world’s population and the planet [21]. The four pillars underpinning healthy and sustainable diets are “nutrition and health”, “affordability and accessibility”, “cultural acceptability”, and “environmental impact” [22]. Modeling studies focused on finding food systems that achieve the best balance between these pillars highlight the need to shift towards more food diverse diets, mainly plant-based diets, while reducing food losses and waste and improving food production, transport, processing, and marketing practices [4,23]. Several organizations, including the EAT Lancet Commission, WHO, the World Wildlife Fund, and the World Resources Institute (WRI), suggest that these diets should be based on diversified flexitarian or territorial food patterns (Mediterranean and New Nordic diets) and dietary patterns with the option to exclude foods of animal origin (e.g., vegetarian, pescatarian, or vegan diets). Healthy diets should comply with the principles described by WHO and FAO, with differentiation between a healthy diet and a healthy dietary pattern [24].

Dietary patterns in the region have been extensively evaluated, especially in the adult population [25,26,27]. It has been observed that there are differences according to socioeconomic level, sex, and educational level. For example, women consume more fruits and vegetables, and drink less alcohol than men; also, the higher the socioeconomic or educational level, the better the quality of food [18,19,20]. However, little is known about the different types of diets consumed.

Recently, our research group published a study that described the different diets and their quality in Latin American and Spain university students. Of the total number of university students evaluated, only 8.8% had a plant-based diet, and these students had better diet quality than the rest of the students [28]. An Argentinian study that assessed lifestyle and dietary adherence showed that vegetarian participants had a higher quality of life and diet compared to omnivorous participants [29].

On the other hand, adopting a sustainable lifestyle involves understanding how lifestyle choices impact the world around us and finding ways for everyone to live better and more sustainable lives. Sustainable living and lifestyles are explicitly mentioned for the first time in the Sustainable Development Goals (Goal 4: Education and Goal 12.8: Responsible Consumption) [30].

However, to our knowledge, the association between dietary patterns and sustainable lifestyles remains unknown. We hypothesize that people following a plant-based pattern have a higher sustainable lifestyle score than people with Western diets. Therefore, the objective of this study is to associate different dietary patterns with sustainable lifestyles in Latin America and Spain.

## 2. Materials and Methods

### 2.1. Research Design

This was an observational, analytical, multicenter, cross-sectional survey study, with a total of 6412 participants. The study was conducted between March 2023 and January 2024. Social networks (Instagram, Facebook, LinkedIn, and Twitter) were used to distribute the questionnaire, and non-probability snowball sampling was used.

### 2.2. Units of Analysis

The inclusion criteria for participants were age 18 years or older, of both sexes, residing in one of the countries participating in the study (Argentina, Bolivia, Chile, Colombia, Costa Rica, Ecuador, El Salvador, Spain, Guatemala, Panama, Paraguay, Peru, Uruguay, and other countries). People who, for medical reasons, had a specific dietary pattern were excluded, for example, subjects with renal or hepatic insufficiency or those receiving enteral nutrition.

A self-administered questionnaire was developed in an online format in the Google Docs interface. The questionnaire was divided into sections:

(1) Sociodemographic background: country of residence, age, sex, educational level, socioeconomic aspects, and place of residence.
(2) Body mass index classification: BMI (kg/m^2^) was determined according to self-perceived weight (in kilograms) and height (in centimeters). For adult BMI classification: underweight <18.5, Normal 18.5 to <25 kg/m^2^, overweight 25 to <30 kg/m^2^, obese ≥30 kg/m^2^ [31].
(3) Feeding patterns.

The dietary patterns proposed were as follows: Western, vegetarian, vegan, ketogenic, Mediterranean, prudent, and paleolithic diets. Each of them was described to facilitate the understanding of the participants, which we based on the study of Moreno et al. [32], so that each participant could identify with the dietary pattern they were currently following (Table S1).

(4) Sustainable Lifestyles Survey

This survey was prepared by experts in the field of nutrition and public health, and was subsequently validated through an analysis of the Content Validity Index (CVI) by calculating Lawshe’s Content Validity Ratio (CVR) [33]. This questionnaire considers sustainable and environmentally friendly lifestyles.

A total of 36 questions were formulated. At this point, each expert assigned each item a score based on three possibilities: that the item is “essential” (1) to evaluate the construct; that it is useful, but dispensable (0); or that it is considered unnecessary (0). The following expression is applied to this evaluation.

(1) $$CVI=\frac{\sum_{i=1}^{M}CVRi}{M}$$

where *n* is the number of experts who agree on the “essential” category (summation of ones) and N is the total number of experts evaluating the content (in this case, 18). Lawshe’s original acceptance criterion for 18 experts was an RVC of 0.56 or higher. The panel of experts consisted of nutritionists, physicians, and agronomists from different regions of Latin America and Spain, with backgrounds in nutrition, ecology, public health, and other fields. After the analysis, all the questions in the questionnaire were accepted, and minimal modifications were made to the wording based on the experts’ suggestions, which proved to be appropriate in improving the clarity and understanding of the questionnaire.

The survey is subdivided into 3 items: the first consists of 15 questions that assess food and shopping; the second consists of 12 questions that assess transportation, recreation, and self-care; and finally, the third consists of 11 questions that assess the environment (Table S2). The total score of the Sustainable Lifestyles Survey is calculated as a continuous variable, with higher scores indicating more sustainable behaviors.

(5) Other variables: We included the description of variables such as smoking habits (asking about frequency and amount of consumption) and physical activity (asking about the frequency of physical activity per week).

Ethics: The study was approved by the Ethics Committee of the Universidad San Sebastián (code 25-23). On opening the survey link, the informed consent form was displayed and on indicating that they agreed to participate, the questions were displayed.

### 2.3. Universe and Sample

The sample size calculation was made considering the data from the last national census of each country. Based on this detail, and considering a 95% confidence level, a sample of 384 participants was estimated for each country, which should be proportional to its population so that the weight of individuals is the same and each country has an equivalent representation for individuals over 18 years of age. This estimation was made using the GRANMO Grandária Mostral Calculator (https://www.datarus.eu/aplicaciones/granmo/, accessed on 6 June 2025).

### 2.4. Data Analysis

Statistical analyses were performed using the Stata 18.0 MP software (StataCorp, College Station, TX, USA). Quantitative variables are presented as means with standard deviations and qualitative variables as frequency and percentage. Linear regression models were used to estimate associations between dietary patterns and sustainability scores. We evaluated key model assumptions, including the normality of residuals, homoscedasticity, and multicollinearity. Given the large sample size, we assumed approximate normality based on the Central Limit Theorem. However, visual inspection of residual plots indicated potential heteroscedasticity. Therefore, we applied robust standard errors to account for non-constant variance. Multicollinearity was assessed using variance inflation factors, and no issues were identified.

All associations are reported as β coefficients, which represent a point increase or decrease in the sustainable lifestyle score, with their corresponding 95% confidence intervals (95% CIs) derived from models using robust standard errors, and a *p* value < 0.05 was considered indicative of statistical significance. Multivariate models were applied to adjust for potential confounding factors. Three adjustment models were used: an unadjusted model (Model 0); a model adjusted for country, sex, and age (Model 1); a model adjusted for smoking and physical activity (Model 2); and a model adjusted for BMI (Model 3 sub-score).

## 3. Results

Table 1 summarizes the demographic and dietary behavioral characteristics and sustainable lifestyles of the 6412 participants. The mean age of the participants was 35.2 years (SD 12.7). The majority of participants identified their dietary pattern as omnivorous (41.5%), followed by the Western diet (21.7%) and the Mediterranean diet (12.7%).

In Table 2, the results of the associations between the different dietary patterns and the total sustainability score are presented. The results indicate that dietary patterns such as vegan (β: 14.90; 95% CI: 9.75–20.05) and lacto egg (β: 12.08; 95% CI: 8.57–15.58) diets are significantly associated with higher sustainability scores compared to an omnivorous diet.

Table 3 details the associations between specific sustainability sub-scores and dietary patterns. The analyses reveal that plant-based diets, such as the vegan (sub-score 1: β: 6.19; 95% CI: 4.43–7.96) diet, are consistently associated with higher levels of sustainability in all areas assessed.

**Figure 1:**
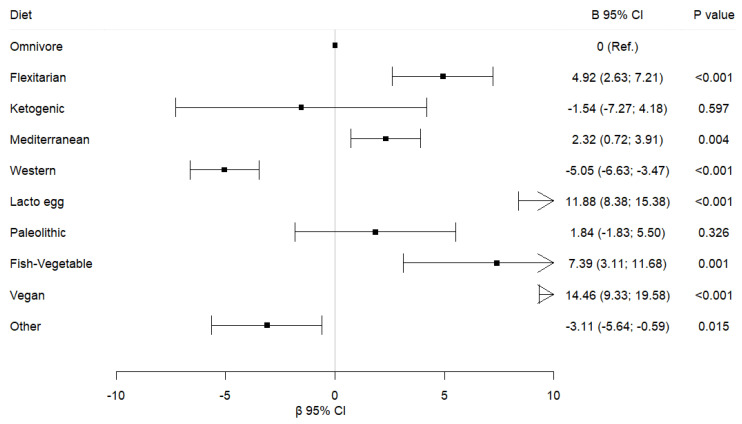
Associations between sustainability scores and dietary patterns.

In contrast, the Western diet shows a significant negative association with sustainability in all subcomponents assessed (Figure 1).

## 4. Discussion

In this study, which examined the associations between different dietary patterns with sustainable lifestyles, it was mainly found that participants who follow plant-based diets, especially people with a vegan diet, exhibit a sustainable lifestyle profile in all three areas evaluated when compared to those following a Western diet. Based on these results, plant-based diets should be encouraged in the region, while respecting and adapting to the characteristics of each country, i.e., national and local food production, food habits and traditions, food security, seasonality, double burden of disease, food culture, and others.

Ensuring a healthy and sustainable diet for all should be a global priority, and to achieve this goal, substantial transformations in the food system are required [34]. Latin American dietary patterns, although they may share common foods such as maize, rice, wheat, beans, potatoes, tomatoes, and avocado, among others, differ from those of other regions of the world [35,36], and due to the vast geographical scope and varying climates, it also boasts a great diversity of foods and culinary preparations, primarily based on plant-based foods [37]. Spain, on the other hand, is dominated by the Mediterranean diet, which includes foods such as fruits and vegetables, whole grains, legumes (pulses), nuts and seeds, and an abundance of olive oil [38]. In general, the contribution of foods of animal origin is lower; in many Latin American dishes, the portion of beef, pork, poultry, or fish can range from 50 to 150 grams, with an average of 75 grams [39]. On the other hand, dairy consumption is close to international recommendations only in countries such as Uruguay, Argentina, Chile, and Costa Rica [40,41].

In the present study, the prevalence of different dietary patterns, particularly plant-based diets, is associated with sustainable lifestyles. Although there is evidence that dietary patterns such as vegetarian and vegan diets are safe and can be recommended to the entire population [42], there may be concerns among health professionals and authorities in the region about recommending such dietary patterns, due to the high prevalence of poverty, food insecurity, chronic malnutrition, and deficiencies in iron, vitamin A, and other nutrients, particularly among infants and young children [43]. Although the data in the region are mixed on these concerns, the poorer population is more susceptible to these nutritional issues. Modeling studies indicate that plant-based, particularly vegan, dietary patterns may lead to vitamin B12 deficiencies [44], since this nutrient is only found in foods of animal origin, thus requiring the consumption of fortified foods and vitamin B12 supplements [44]. Iodine, which is present in both animal-derived foods and iodized salt, can also be a concern, as not consuming foods fortified with iodine or consuming artisanal salts could mean a risk of a lower intake of this mineral [45].

In contrast, the study results show that the Western diet, high in sugars, saturated fats, and salt, was associated with lower sustainable lifestyle scores. The high consumption of ultra-processed products, characteristic of Western dietary patterns, is prevalent in the region in general, with Mexico, Chile, and Argentina being the countries with the highest consumption. In particular, Latin American countries lead the global consumption of sugar-sweetened beverages [46,47,48]. The consumption of sugar-sweetened beverages has been associated with multiple health issues such as obesity, metabolic syndrome, diabetes, certain types of cancer, and dental cavities. Additionally, their packaging can generate significant amounts of waste and environmental pollution [49]. In addition to ultra-processed foods, some diets in the region also show a high consumption of home-made fried foods [50], street food, and informal sales of mainly fried foods [51], which is also associated with negative health effects and pollution, since these oils are poured directly into sewers, generating solidification and saponification [52], polluting rivers, lakes, and the sea.

On the other hand, a study in women showed that those with higher scores on the dietary health and sustainability index had a lower risk of depression, anxiety, and psychological distress [53]. Another study conducted in children and adolescents determined the main contributors of greenhouse gases (GHGs) of dietary origin and land use (LU), determining these were meat products (GHG: 25.6%; LU: 32.8%), dairy products (22.2%; 17.7%), and sweets and pastries (14.0%; 14.3%); as well as soft drinks (24.3%), and vegetables and fruits (17.7%) [54].

A modeling study comparing seven countries demonstrated that changes in dietary patterns toward longevity-optimized or vegan diets would result in substantial gains in life expectancy across all ages and countries. These changes involve more frequent consumption of whole grains, legumes, and nuts, while reducing red/processed meats, sugars, and sugar-sweetened beverages [55].

Studies conducted among Latin American university students have shown that 8.8% followed plant-based diets, with 50% of them being ovo-dairy-vegetarian. These students exhibited the best adherence to healthy dietary patterns, especially the vegan students [28]. Another study conducted in Chile during the pandemic showed that individuals following plant-based diets consumed legumes more frequently, surpassing the recommendations of the Chilean dietary guidelines, and also prepared a greater variety of legume-based dishes [56]. However, for vegetarians and vegans, the primary source of information is not nutritionists or health professionals, but rather the internet [57], which increases the risk of encountering erroneous or low-quality information.

A panel has been designed and developed to enhance healthy and sustainable food choices, aiming to meet sustainability goals from a pre-consumption perspective [55]. However, further research is needed on the relationship between dietary patterns and sustainability behaviors within the population. This will inform the design of public policies aimed at improving population health while preventing the deterioration of the planet. These actions include, for example, the preservation of agrifood biodiversity, the revaluation of underutilized ancestral crops with nutritional potential, and the implementation of nature-based solutions to strengthen the link between the bioeconomy, human health, and planetary health. The region is moving in this direction, as evidenced by the updating of national food guides, now integrating the food system approach, which also considers sustainable forms of production and access to healthy diets.

Although the concept of a healthy diet has been described, there is still controversy, as no single pattern exists. In the case of Latin America and Spain, the patterns of healthy and sustainable diets have not been fully described, although each country has its unique food consumption practices. Many Latin American countries are exporters of fresh foods to the world. However, each country has typical foods or food groups that nutritionally enrich their populations, and adjusting these to international recommendations presents a major challenge for nutrition professionals and health authorities in the region [58]. In addition to promoting a healthy diet, we must also work to ensure that it is safe, secure, and aligned with sustainability guidelines.

Among the weaknesses of the study, it can be mentioned that, despite having a large number of participants and countries, they are not a representative sample of the general population. Additionally, the study design, based on a cross-sectional survey, does not allow us to speak of causality, but only of association. Another limitation is that the proportion of women is significantly higher than that of men; however, this appears to be a consistent finding in online food studies, where women are more likely to respond [59,60]. Finally, this was self-reported. This approach may introduce recall bias and social desirability biases, as participants could overestimate the healthiness or sustainability of their choice. Among the strengths of this study, it is worth mentioning that validated surveys were used, and that the results are presented from a significant number of countries.

## 5. Conclusions

This multicenter study shows that plant-based diets, particularly vegan diets, are associated with more sustainable lifestyles in Latin America and Spain. These findings underscore the importance of promoting dietary patterns that are both healthy and environmentally responsible. However, cultural, economic, and nutritional contexts must be considered when implementing dietary recommendations. Public policies should support the development of sustainable, inclusive, and safe food environments. Future longitudinal studies are needed to better understand the determinants of sustainable food practices and their long-term impact on human and planetary health.

## Figures and Tables

**Table 1:** Sample characteristics.

N	6412
Age, mean (SD)	35.21 (12.67)
Which of the following dietary patterns best describes you?	
1. Flexitarian	505 (7.9%)
2. Keto	78 (1.2%)
3. Mediterranean	817 (12.7%)
4. Western	1389 (21.7%)
5. Lacto egg	220 (3.4%)
6. Paleolithic	154 (2.4%)
7. Pesco-vegetarian	101 (1.6%)
8. Omnivorous	2658 (41.5%)
9. Vegan	75 (1.2%)
10. Other	415 (6.5%)
Which country are you from?	
1. Argentina	740 (11.5%)
2. Bolivia	316 (4.9%)
3. Chile	753 (11.7%)
4. Colombia	561 (8.7%)
5. Costa Rica	433 (6.8%)
6. Ecuador	410 (6.4%)
7. El Salvador	375 (5.8%)
8. Spain	654 (10.2%)
9. Guatemala	225 (3.5%)
10. Honduras	7 (0.1%)
11. Mexico	511 (8.0%)
12. Nicaragua	7 (0.1%)
13. Panama	207 (3.2%)
14. Paraguay	291 (4.5%)
15. Peru	413 (6.4%)
16. Puerto Rico	5 (0.1%)
17. Uruguay	480 (7.5%)
18. Venezuela	24 (0.4%)
What is your gender?	
1. Female	5019 (78.3%)
2. Male	1376 (21.5%)
3. Other	17 (0.3%)
Did you smoke cigarettes?	
1. No	4847 (75.6%)
2. Yes	1565 (24.4%)
Do you currently get at least 150 minutes of physical activity each week?	
1. No	3506 (54.7%)
2. Yes	2906 (45.3%)
BMI, mean (SD)	25.33 (5.20)

**Table 2:** Associations between sustainability score and dietary patterns.

	Model 0		Model 1		Model 2	
Diet	β, 95% CI	*p*-Value	β, 95% CI	*p*-Value	β, 95% CI	*p*-Value
Omnivorous	Ref.		Ref.		Ref.	
Flexitarian	6.12 (3.54; 8.71)	<0.001	5.24 (2.93; 7.56)	<0.001	5.24 (2.93; 7.56)	<0.001
Keto	−0.86 (−6.83; 5.11)	0.777	−1.04 (−6.72; 4.63)	0.719	−1.04 (−6.72; 4.63)	0.719
Mediterranean	3.58 (1.93; 5.22)	<0.001	2.95 (1.37; 4.53)	<0.001	2.95 (1.37; 4.53)	<0.001
Western	−6.34 (−8.03; −4.66)	<0.001	−5.63 (−7.20; −4.06)	<0.001	−5.63 (−7.20; −4.06)	<0.001
Lacto egg	10.79 (6.82; 14.75)	<0.001	12.08 (8.57; 15.58)	<0.001	12.08 (8.57; 15.58)	<0.001
Paleolithic	1.38 (−2.39; 5.15)	0.473	1.61 (−2.13; 5.34)	0.398	1.61 (−2.13; 5.34)	0.398
Pesco-vegetarian	6.70 (1.80; 11.60)	0.007	8.27 (4.01; 12.53)	<0.001	8.27 (4.01; 12.53)	<0.001
Vegan	11.64 (6.09; 17.19)	<0.001	14.90 (9.75; 20.05)	<0.001	14.90 (9.75; 20.05)	<0.001
Other	−4.39 (−7.05; −1.72)	0.001	−3.71 (−6.23; −1.20)	0.004	−3.71 (−6.23; −1.20)	0.004

Model 0, unadjusted; Model 1, country, sex, and age; Model 2, country, sex, age, smoking, and physical activity.

**Table 3:** Associations between sustainability sub-scores and dietary patterns.

	Food and Shopping		Transport, Recreation, and Self-Care		Environment	
Diet	β, 95% CI	*p*-Value	β, 95% CI	*p*-Value	β, 95% CI	*p*-Value
Omnivorous	Ref.		Ref.		Ref.	
Flexitarian	2.56 (1.65; 3.46)	<0.001	0.75 (−0.09; 1.58)	0.081	1.62 (0.77; 2.47)	<0.001
Keto	−0.80 (−3.20; 1.60)	0.513	−0.16 (−2.17; 1.85)	0.875	−0.58 (−2.75; 1.59)	0.600
Mediterranean	1.38 (0.73; 2.03)	<0.001	0.09 (−0.50; 0.69)	0.760	0.84 (0.22; 1.46)	0.008
Western	−2.45 (−3.09; −1.81)	<0.001	−1.55 (−2.12; −0.98)	<0.001	−1.05 (−1.66; −0.44)	0.001
Lacto egg	5.77 (4.35; 7.18)	<0.001	2.59 (1.37; 3.81)	<0.001	3.52 (2.20; 4.85)	<0.001
Paleolithic	0.20 (−1.28; 1.69)	0.790	0.74 (−0.59; 2.08)	0.274	0.89 (−0.57; 2.35)	0.233
Pesco-vegetarian	3.69 (1.98; 5.41)	<0.001	1.15 (−0.43; 2.73)	0.155	2.55 (0.93; 4.17)	0.002
Vegan	6.19 (4.43; 7.96)	<0.001	3.28 (1.43; 5.13)	0.001	4.99 (2.90; 7.07)	<0.001
Other	−1.39 (−2.46; −0.31)	0.011	−0.69 (−1.59; 0.21)	0.131	−1.04 (−2.02; −0.05)	0.039

Fully adjusted model, adjusted for country, sex, age, smoking, physical activity, and body mass index.

## Data Availability

The original contributions presented in this study are included in the article/Supplementary Material. Further inquiries can be directed to the corresponding author.

## Supplementary Materials

The following supporting information can be downloaded at https://www.mdpi.com/article/10.3390/foods14122065/s1: Text S1: Dietary patterns and food consumption; Table S1: Healthy and sustainable activities.
