# Supplementary material for: Dietary Patterns and Sustainable Lifestyles: A Multicenter Study from Latin America and Spain
Source: Foods. 2025 Jun 11;14(12):2065. doi: 10.3390/foods14122065 (PMC12192246; doi:10.3390/foods14122065)
Supplement: Supplementary file 1 [file foods-14-02065-s001.zip › foods-3611706-supplementary.pdf]

Supplementary Text S1.

DIETARY PATTERNS AND FOOD CONSUMPTION

Please choose the alternative below that most closely identifies with your usual food consumption.

Which of the following dietary patterns best describes you?

a) Western diet: Omnivorous diet that usually includes a high consumption of animal-based foods, highly processed foods and a lower than recommended consumption of plant-based foods.

b) Prudent diet: An omnivorous diet characterised by a prudent or moderate consumption of all foods, avoiding over- and under-consumption, according to the dietary guidelines of your country.

c) Flexitarian diet: An omnivorous diet that includes high amounts of plant-based foods, moderate amounts of poultry, dairy and fish, and low amounts of red meat, highly processed foods and added sugar.

d) Mediterranean diet: Omnivorous diet, characterised by high consumption of vegetables, fruits, nuts, seeds, olives, olive oil and fish.

e) Vegan diet: Characterised by consuming only foods of plant origin.

f) Ovo-dairy-vegetarian diet: Characterised by the consumption of plant foods, eggs and dairy products.

g) Fish-vegetarian diet: Characterised by the consumption of plant foods, fish, eggs and dairy products.

h) Keto diet: Diet characterised by being high in fat and low in carbohydrates.

i) Paleolithic diet: Diet is characterised by being rich in meats, vegetables and seeds, but excludes dairy, cereals and legumes.

j) Other diet.

Supplementary Table S1

Healthy and Sustainable Activities

The options below are 5, ranging from Always to Never.

Food and Shopping

	Always	Almost always	Sometimes	Rarely	Never
Prefer to buy food produced near your city or locally grown					
Prefer to buy from markets or food fairs rather than supermarkets					
Prioritize purchasing seasonal fruits and vegetables					
Check your food to prevent spoilage					
Check the expiration dates of packaged food to avoid it expiring					
Prioritize consuming plant-based protein sources such as legumes (lentils, chickpeas, beans, etc.)					
Avoid disposing of large amounts of cooking oil in the dishwasher/sink when used in large quantities in culinary preparations					
Limit the consumption of red meat such as beef, pork, and others					
Limit the consumption of processed meats (sausages, chorizo, etc.)					
Prefer foods with less packaging					
Choose to eat organic food					
Frequently use food leftovers (in other culinary preparations or other domestic uses)					
When serving your plate, take only what you will eat					
Store freshly cooked (hot) food in the fridge					
Buy bulk foods such as legumes, nuts, fruits, vegetables, etc					

Transport, Recreation, and Self-care

	Always	Almost always	Sometimes	Rarely	Never

For short distances (less than 10 blocks), prefer walking over using public transportation or a car					
For trips under 1 hour, prefer using a bicycle rather than public transportation or a car					
For long-distance travel within or outside the country, prefer land transportation whenever possible					
When camping, properly dispose of waste or take it home					
Concerned about ensuring your vehicle's or someone else's tires have the correct air pressure to improve vehicle handling and save fuel					
Prioritize the use of "cruelty-free" products (free from animal abuse)					
Aim to shower in less than 5 minutes					
Turn off the water tap while brushing your teeth					
Evaluate whether you truly need new electrical and electronic devices before purchasing them					
Prioritize purchasing appliances with an A rating (the most energy-efficient)					
Use air conditioning responsibly, avoiding the use of climate control equipment (air conditioning), preferring light or warm clothing, as appropriate, to maintain a comfortable body temperature					

Environment

	Always	Almost always	Sometimes	Rarely	Never
Take your own bag when shopping					
Avoid buying products with excessive plastic					
Recycle or reuse your clothes/shoes for new seasons					
Avoid using paper or plastic straws to drink water/beverages/juices					
Replace plastic bottles with glass bottles for carrying water					
Sort waste for recycling					
When disposing of used masks, cut the elastic bands					
Collect batteries and take them to recycling centers					

Maximize natural light and ventilation					
Opt to receive receipts and invoices online					
Turn off the TV when you are not watching					

Material Suplementar - Questionário
Atividades Saudáveis e Sustentáveis
As opções disponíveis abaixo são 5, variando de Sempre a Nunca

Alimentação e compras

	Sempre	Quase sempre	Às vezes	Quase nunca	Nunca
Prefere comprar alimentos produzidos perto da sua cidade ou cultivados localmente					
Prefere comprar em mercados ou feiras de alimentos do que no supermercado					
Privilegia a compra de frutas e vegetais da estação					
Verifica os seus alimentos para evitar que se estraguem.					
Verifica as datas de validade dos alimentos embalados para evitar o vencimento.					
Prioriza o consumo de fontes proteicas de origem vegetais como leguminosas (lentilha, grão de bico, feijão, etc.)					
Quando utiliza óleos em grande quantidade nas suas preparações culinárias evita descartá-lo na máquina de lavar loiça/pia?					
Limita o consumo de carne vermelha como: bovina, suína, outras.					
Limita o consumo de carnes processadas (salsicha, chouriço, etc.)					
prefere alimentos com menos embalagens.					
Escolhe comer alimentos biológicos					
Costuma utilizar as sobras de comida (em outras preparações alimentares ou outro uso doméstico)					
Ao preparar o seu prato, serve-se do que realmente vai comer.					
Coloca alimentos recém cozinhados (quentes) no frigorífico.					
Compra alimentos a granel, como leguminosas, frutos gordos, frutas, vegetais, etc.					

Transporte, recreação e autocuidado

	Sempre	Quase sempre	Às vezes	Quase nunca	Nunca
Para distâncias curtas (menos de 10 quarteirões) prefere caminhar do que usar transporte público ou usar carro					
Para distâncias inferiores a 1 hora, prefere usar bicicleta a usar transporte público ou carro					
No caso de viajar longas distâncias dentro ou fora do país, prefere o transporte terrestre sempre que possível					
Quando vai acampar, descarta o lixo de maneira adequada ou leva-o para casa.					
Está preocupado que os pneus do seu veículo ou de alguém próximo tenham a pressão de ar adequada para melhorar a movimentação do veículo e economizar combustível.					
Privilegia o uso de produtos "cruelty free" (livres de abuso de animais)					
Procura tomar banho em menos de 5 minutos					
Fecha a torneira da água enquanto escova os dentes.					
Antes de comprar novos equipamentos elétricos e eletrónicos, avalia se realmente precisa deles					
Privilegia a compra de eletrodomésticos com etiqueta A (o que consome menos energia).					
Utiliza o ar condicionado de forma responsável, evitando o uso de equipamentos de condicionamento (ar condicionado), privilegiando o uso de roupas leves ou quentes, conforme o caso, para manter uma temperatura corporal adequada.					

Meio ambiente

	Sempre	Quase sempre	Às vezes	Quase nunca	Nunca
Quando vai às compras leva o seu próprio saco					
Evita comprar produtos que trazem muito plástico					

Recicla ou reutiliza as suas roupas/sapatos para as novas estações					
Evita usar palhinhas de papel ou plástico para beber água/bebidas/sumos					
Para transportar água, substitui garrafas de plástico por garrafas de vidro					
Realiza separação de lixos					
Ao descartar as máscaras usadas, corta os elásticos.					
Junta as pilhas e baterias e leva-as para a reciclagem.					
Aproveita ao máximo a luz e ventilação naturais					
Privilegia que lhe enviem recibos e faturas on-line.					
Desliga a TV quando não está assistindo					

Material Suplementario_Cuestionario
Actividades Saludables y sostenible
Las opciones disponibles son 5, variando de siempre a nunca

Alimentación y compras

	Siempre	Casi Siempre	A veces	Casi nunca	Nunca
Prefieres comprar alimentos que se producen cerca de tu ciudad o cultivados localmente.					
Prefieres comprar en mercados o ferias de alimentos que en el supermercado					
En el caso de frutas y verduras privilegias comparar las de temporada					
Revisas tus alimentos para evitar que se pudran.					
Revisas las fechas de vencimiento de los alimentos envasados para evitar que caduquen.					
Priorizas el consumo de proteínas vegetales tales como legumbres (lentejas, garbanzos, frejol, etc.).					
Cuando ocupas aceite en cantidades elevadas en tus preparaciones, evitas desecharlo por el lavaplatos.					
Limitas el consumo de carnes rojas frescas como: vacuno (res), cerdo, otro.					
Limitas el consumo de carne procesada (salchichón, salchicha, chorizo, etc.)					
Prefieres alimentos con menos embalaje .					

Eliges el consumo de alimentos orgánicos.					
Sueles utilizar la comida sobrante (en otras preparaciones alimentarias u otro uso doméstico)					
Al momento de preparar el plato, sirves lo que realmente comerás.					
Introduces alimentos recién cocinados (calientes) al refrigerador).					
Compra a granel alimentos como legumbres, frutos secos, frutas, verduras, etc.					

Transporte, recreación y autocuidado

	Siempre	Casi Siempre	A veces	Casi nunca	Nunca
Para distancias cortas (menos de 10 cuadras) prefieres caminar que usar trasporte público o usar en automóvil/carro					
Para distancias de menos de 1 hora prefieres utilizar bicicleta que usar trasporte público o automóvil/carro/moto					
En caso de viajar grandes distancias ya sea dentro del país o al extranjero, prefieres el transporte terrestre siempre que sea posible					
Cuando vas de camping, eliminas la basura correctamente o la traes de vuelta a tu casa.					
Te preocupa de que los neumáticos de tu vehículo o de algún cercano, tengan la presión de aire adecuada para mejorar el desplazamiento del vehículo y ahorrar gasolina.					
Privilegias el uso de productos "cruelty free" (libre de maltrato animal)					
Cuando te duchas tratas de hacerlo en menos de 5 minutos					
Cuando te cepillas los dientes cierras la llave/grifo del agua					
Antes de comprar nuevos equipos eléctricos y electrónico, evalúas si realmente lo necesitas					
Privilegias la compra de electrodomésticos con etiqueta A (es la que consume menos energía).					
Usas climatización de forma responsable , evitando el uso de equipos de acondicionamiento (aire acondicionado), privilegiando usar ropa liviana o abrigada,					

según corresponda, para mantener una temperatura corporal adecuada.					

Medio ambiente

	Siempre	Casi Siempre	A veces	Casi nunca	Nunca
Cuando vas a comprar llevas tu bolsa reciclada					
Evitas comprar productos que traen mucho plástico.					
Reciclas o reutilizas tu ropa/calzado para nuevas temporadas					
Evitar usar pajitas (popote, sorbete) de papel o plástica cuando toma agua/bebidas/jugos					
Para transportar agua reemplazas las botellas de plástico por las de vidrio					
Separas la basura en papeles, cartones, otros					
Cuando eliminas (tiras a la basura) las mascarillas usadas, les cortas los elásticos.					
Las pilas/baterías, las juntas y las llevas a un centro de reciclaje					
Aprovechas al máximo la luz y ventilación natural					
Privilegias que te den las boletas o facturas por internet.					
Apagas la televisión cuando no la estás viendo					
